# Medical student perceptions of assessments of clinical reasoning in a general surgery clerkship

**DOI:** 10.1186/s12909-024-05184-w

**Published:** 2024-03-01

**Authors:** Rachael Tolsma, Saad Shebrain, Shamsi Daneshvari Berry, Lisa Miller

**Affiliations:** 1 Department of Orthopaedic Surgery, University of Wisconsin-Madison, 1685 Highland Ave, Madison, WI 53705 USA; 2 Department of General Surgery, Western Michigan University Homer Stryker MD School of Medicine, Kalamazoo, MI USA; 3 Department of Biomedical Informatics, Western Michigan University Homer Stryker MD School of Medicine, Kalamazoo, MI USA

**Keywords:** Oral examination, General surgery clerkship, Clinical reasoning, Medical student perception, Assessment

## Abstract

**Background:**

Components factoring into general surgery clerkship grades vary by institution, and while evaluators attempt to remain unbiased when evaluating medical student performance, subjectivity and implicit bias remain an issue. Our institution recently implemented a case-based structured oral examination to provide the general surgery clerkship director objective insight into students’ clinical reasoning skills. We hypothesized that medical students believe this exam, along with graded clinical documentation and the Observed Standardized Clinical Encounter (OSCE), are fair assessments and increase students’ awareness of their clinical reasoning skills.

**Methods:**

A survey was sent to third-year medical students in the classes of 2023 and 2024 at our institution who had completed their general surgery clerkship. Students rated five grading assessments (i.e., preceptor evaluations, the oral examination, clinical documentation, the OSCE, and the shelf exam) on fairness and the ability of the assessment to give them insight into their clinical reasoning on a five-point Likert scale 1–5 (with 1 = Strongly Agree, 5 = Strongly Disagree).

**Results:**

One hundred and ten of 162 (67.9%) students responded to the survey. The shelf examination was the most highly regarded assessment tool followed by the oral examination. Seventy-three percent agreed or strongly agreed that the oral exam was a fair assessment, and 80% agreed or strongly agreed that it gave them insight into their clinical reasoning skills. Alternatively, only 41.8% of students agreed or strongly agreed that preceptor evaluations were fair assessments and 42.7% agreed or strongly agreed that it gave them insight into their clinical reasoning.

**Conclusions:**

Third-year medical students on a general surgery clerkship favor the shelf examination and a case-based oral examination over other assessment tools regarding fairness and perception of their clinical reasoning. This type of examination can provide general surgery clerkship directors with additional objective data to assess medical students more fairly and improve students’ clinical reasoning.

## Background

Third-year clinical grades are one of many components that factor into the competitiveness of a medical student’s application for residency. General surgery program directors cite clinical grades as essential in deciding which medical students to interview for residency positions and which to rank in the Match [1]. Assessments to grade medical students on the general surgery clerkship typically include preceptor evaluations, Observed Standardized Clinical Encounters (OSCEs), clinical documentation, internally written examinations, the National Board of Medical Examiners (NBME) standardized examination (shelf exam), and computer case simulators [2]. There are advantages and disadvantages to each method of assessment of knowledge and clinical reasoning, necessitating multiple modes of assessment to capture the clinical acumen of each student completely.

Lower grades may impact students’ competitiveness when applying for residency, and ultimately limit their matriculation into the physician workforce. It has been reported that patients prefer physicians of the same race or ethnicity and that female patients have better outcomes when treated by female surgeons [3, 4]. To better care for our diverse patient populations, we need a physician workforce with similar diversity.

Several assessment methods can be subjective and may be prone to bias that disproportionately impacts students. Literature shows that those who identify as underrepresented in medicine (URiM) are less likely to obtain honors in core clerkships than their white counterparts, even when controlling for other factors [5, 6]. One institution found that disparities existed in both the assessments administered and the clinical environment, leading to disparity in final grades [7, 8]. The need for a diverse physician workforce, combined with the current biases in clerkship grading, necessitates us to dismantle barriers encountered by URiM students and develop clerkship grading systems that are equitable.

Structured, case-based oral examinations have the potential to make grading in general surgery clerkships more equitable and can feasibly be included in a general surgery clerkship curriculum. Traditional assessment methods, such as preceptor evaluations and written clerkship evaluations, are especially susceptible to subjectivity and have been shown to correlate poorly with standardized examination scores, and therefore may disproportionately impact those underrepresented in medicine [6, 9, 10]. The standardization of a structured, case-based oral examination allows for increased transparency of grading, increased objectivity, and decreased opportunity for bias. Disadvantages of oral examinations include their development and historically poor interrater reliability, particularly with students who performed poorly [11]. The process of standardization decreases the subjectivity of the oral examination, and improves inter-examination reliability [7]. Research is ongoing on how to best develop structured oral examinations and ensure validity [12]. Once established, faculty have been shown to favor an oral examination as it allows them insight into several qualities not assessed in other examinations [13].

While structured oral exams can offer a similar evaluation of overall knowledge as other assessment methods, evaluators can observe additional traits important to clinical reasoning, such as focus, organization, thoroughness, pacing, and decisiveness, that other assessments do not measure [14]. Clinical reasoning is a vital skill that clerkship directors must evaluate during the general surgery clerkship. Clinical reasoning in medical school is defined as the process by which medical students can gather and interpret data to diagnose and treat patients and is required for several of the Association of American Medical College’s (AAMC) core entrustable professional activities (EPAs) that all students must meet upon graduation from medical school [15, 16]. In addition to providing the evaluator with crucial insight into student performance, oral examinations are favored by medical students. Caldwell et al. demonstrated that a structured oral examination decreased racial grading discrepancy and that URiM students felt it was a fair assessment method [7]. Despite being favored by students and faculty, there is currently a lack of substantial research investigating the oral examination as a standardized, objective assessment method in the general surgery clerkship.

To eliminate as much bias as possible and to increase clerkship director and medical student insight into clinical reasoning abilities, our institution has implemented a structured case-based oral examination on the general surgery clerkship. Our institution uses the oral exam, graded clinical notes, and the traditional OSCE to compile objective evidence for each student’s level of clinical reasoning to guide the clerkship director’s preceptor evaluation. When combined with other preceptor evaluations and the NBME exam, we feel this grading matrix gives the clerkship director a comprehensive view of the student’s overall performance, clinical knowledge, and clinical reasoning. We hypothesized that medical students view the OSCE, oral examination, NBME subject exam, and patient documentation as fair assessment tools of clinical knowledge and that they increase medical students’ awareness of their clinical reasoning skills. We also hypothesize that URiM students and female students will view more objective assessments, such as the OSCE, oral examination, NBME exam, and patient documentation as more fair than subjective assessments, such as preceptor evaluations. While medical student perception does not necessarily correlate with performance, medical students’ opinion should be heard. If our hypothesis is supported, this would provide support for using several objective assessments to evaluate student knowledge and clinical reasoning to help grade students in the general surgery clerkship. 

## Materials and methods

### Undergraduate education at our institution

Our institution implements a system based preclinical curriculum for the first 2 years of medical school. Students then rotate through six core clerkships: family medicine, general surgery, internal medicine, pediatrics, psychiatry/neurology, and obstetrics and gynecology, throughout their third year. In their fourth year, they are required to take emergency medicine, an intensive care rotation, and a hospital-based inpatient rotation along with clinical and non-clinical electives.

### Oral examination

Students are given a patient and chief concern and then direct the examination themselves by asking questions about the history of present illness, subjective findings, and objective findings from physical exam, labs, and imaging. From their findings based on the questions they asked, they must give a differential diagnosis and treatment plan. Every relevant question a medical student could ask is prewritten to provide as similar of an experience as possible for all students to eliminate bias.

This examination occurs during the last week of the clerkship. Each student is given two patient cases and allotted 30 minutes total. The students on each rotation are given the same cases, but cases are rotated between rotations to ensure case integrity. The cases are of similar difficulty and based on a list of common general surgery diagnoses, all of which students are required to experience in the clinical setting over the course of their clerkship. Questions and answers were developed by the clerkship director and are based on deidentified real patient cases.

Students are graded by the clerkship director from a standardized template that was approved by the surgical faculty. They are graded on their ability to identify and manage different common general surgery diagnoses and are provided written feedback on their performance afterwards. This feedback includes a statement about their overall performance and then specific feedback on positive parts of their performance (i.e. good history questions, logical progression through case, appropriate management plan) as well as any concerns or ways to improve (i.e. more detailed history, more aggressive resuscitation).

### OSCE

The OSCE occurs during the last week of the clerkship. Students are given 15 minutes for the patient encounter, where they must obtain a history and perform a physical exam as they deem necessary. Students are graded on this portion by the standardized patient with a 40-point communication checklist. Immediately after the encounter, students have 20 minutes to write a history and physical note including subjective, objective, assessment, and plan portions. Scores for the OSCE note are graded by surgery faculty based on a rubric standardized across all clinical clerkships at our institution with more emphasis placed on the assessment and plan portion.

### Clinical documentation

Students submit three patient notes, at least one of which must be a history and physical and at least one of which must be submitted prior to the midclerkship meeting. The clerkship director provides feedback and takes into consideration the improvement of the notes over the course of the clerkship.

### Preceptor evaluations

Students provide names of midlevel providers, residents, and attendings with whom they have worked over the clerkship. These individuals include faculty surgeons, both core academic and community faculty, surgical residents and midlevel providers on the services. These preceptors are then sent a standardized form electronically and asked to rate students on a scale from early developing, progressing towards later developing, later developing, progressing towards entrustable, and entrustable for both clinical performance and interprofessional skills. There is also an option for unable to assess if the preceptor was not able to observe a certain skill. Finally, there are spaces for free text on things students did well and areas for improvement. All of these individuals work with students routinely and have been given instruction on how to complete these assessments objectively. The clerkship director is included in this group and completes evaluations on all students at the end of the clerkship taking into account the performance on the clinical documentation, OSCE, and oral examination.

### NBME

At the end of the clerkship, students sit for the timed 110-question standardized NBME examination with 90 seconds per question. This is graded based on national averages, with greater than the 5th percentile required to pass, 50th to 69th percentile required to high pass, and greater than or equal to the 70th percentile required to achieve honors on the examination in the clerkship.

### Final grades

Our clerkships are graded as fail, pass, high pass, and honors. To achieve honors, a student must honor clinically, based on the preceptor evaluations as outlined above, and obtain honors on the NBME shelf examination. Likewise, to obtain a high pass, a student must obtain at least high pass clinical distinction and a high pass on the NBME shelf examination. A student’s overall grade is determined by the lowest grade they receive, for example, if a student were to obtain clinical honors, but a high pass on the NBME exam, their final grade would be a high pass. Once all evaluations have been completed, they are compiled and averaged to complete the summative assessment including comments for the MSPE (Dean’s letter).

### Participants

After completion of their third-year core general surgery rotation, the third-year medical students in the class of 2023 and 2024 at our institution were sent a survey evaluating their perceptions of the assessments used for grading the clerkship. This observational study took place at Western Michigan University Homer Stryker MD School of Medicine from the summer of 2021 to summer of 2023. Inclusion criteria were students who had completed the general surgery clerkship in its entirety, including all assessments previously mentioned. Exclusion criteria were those who completed the general surgery clerkship without an oral examination. Participation was completely voluntary and participants were de-identified, therefore informed consent was deemed unnecessary by the Western Michigan University Homer Stryker MD School of Medicine IRB (WMed-2022-0851, delivered on 1/18/2022). They received the survey upon completion of their general surgery clerkship.

### Questionnaire

Each of the five assessments used in our clerkship: preceptor evaluations, the oral examination, clinical documentation, the OSCE, and the shelf exam, plus the written feedback from their performance on the OSCE, clinical documentation, and the oral examination were rated on fairness, defined as impartial and just, and insight into clinical reasoning. Students rated each on a five-point Likert scale 1–5, ranking (1) strongly agree, (2) agree, (3) neutral, (4) disagree, and (5) strongly disagree. Students also reported demographic information, including age, gender identity, whether they considered themselves URiM, and whether they were planning to pursue a surgical specialty. The questionnaire underwent two cognitive interviews with sample students run by a database specialist to ensure survey-taker comprehension. To reduce nonresponse bias, students were provided the survey with their end of clerkship evaluations and sent emails containing the link as well as reminder emails. Questions were worded with neutral phrases, no leading questions, and students were aware their answers would be deidentified in attempt to reduce response bias. Per IRB instruction, informed consent was waived as participants were deidentified and this disclaimer was included at the beginning of the questionnaire. Surveys were sent electronically via REDCap software and was managed by our data manager of our virtual data warehouse and department of biostatistics. Most surveys were completed directly after the general surgery clerkship (two subgroups were sent the survey months after it had been completed). The questionnaire is included as supplementary data.

### Statistical analysis

For the overall summary of the data, ratings of strongly agree and agree were combined, as were disagree and strongly disagree ratings. However, for the remaining, the full Likert scale was used. To test if the distribution of Likert answers by medical students on fairness and insight into clinical reasoning differed across pursuit of surgical specialty, URiM, and gender, a Wilcoxon rank-sum test was conducted. The null hypothesis was that the distributions of median difference between the two groups are zero. A Bonferroni adjustment (alpha = 0.05/12 = 0.0042) was made to account for the high number of tests. To determine whether there was a difference in medical student perception of fairness and insight into clinical reasoning when comparing each assessment to the other assessments, we performed a pairwise analysis using Wilcoxon Signed-Rank test. This was used to do 10 pairwise comparisons and was implemented using the full spectrum of data (Likert 1–5). The null hypothesis was that the median difference between the paired data is zero (there is no difference in fairness between the two assessments), with significance with a Bonferroni adjustment at 0.05/10 = 0.005 to account for the large number of tests. Similarly, for insight into clinical reasoning, a pairwise analysis using Wilcoxon Signed-Rank test was used to do 21 pairwise comparisons and was implemented using the full spectrum of data. The null hypothesis was that the median difference between the paired data is zero (there is no difference in clinical reasoning between the two assessments), with significance at 0.05/21 = 0.0024. SAS v9.4 was used for all analyses. The sample was a convenience sample; however, effect sizes were calculated for analyses using Cliff’s Delta [17]. Missing data was excluded for each analysis.

## Results

### Demographics

One hundred and ten of the 162 third-year medical students who completed the general surgery clerkship for the class of 2023 and 2024 responded to the survey (67.9% response rate). The cohort was made of 44% men and 54% women, 18.5% identified as underrepresented in medicine, and 25% were planning on pursuing a surgical specialty. The average age of participants was 26.31 years old. Seven surveys did not contain URiM status, two did not contain pursuit of specialty, and five did not include gender information and were excluded from analysis.

### Assessment fairness

Students were asked whether they thought each of the five assessments (i.e., preceptor evaluations, the oral examination, clinical documentation, the OSCE, and the shelf exam) was a fair assessment and rated their perceptions on the five-point Likert scale. First, the preceptor evaluations were the most poorly regarded assessment, with 41.8% students strongly agreeing or agreeing they were fair and 28.1% disagreeing or strongly disagreeing. Second, the standardized shelf examination was found to be the most well-regarded assessment, with 76.4% of students strongly agreeing or agreeing that it was a fair assessment and 7.3% disagreeing or strongly disagreeing. The other three assessments, clinical documentation, the OSCE, and the oral examination, received 62.7%, 60.0%, and 73.6% of students strongly agreeing or agreeing they were fair, respectively (Table 1). To determine which assessments medical students considered fairer than others, we performed pairwise comparisons of every assessment. There were significant differences for perceptions of fairness with all comparisons of the preceptor exam, with the preceptor exam scoring as less fair for all comparisons. Additionally, the OSCE exam and shelf exam were significantly different, with the shelf exam having more medical students agreeing that it was fair (Table 2).

**Table 1:** Medical student perceptions of assessment fairness

Assessment	Strongly agree/ Agree	Neutral	Disagree/Strongly disagree
Preceptor evaluations	46 (41.82%)	24 (26.32%)	40 (28.07%)
Oral examination	81 (73.64%)	16 (14.55%)	13 (11.82%)
Clinical documentation	69 (62.73%)	28 (28.00%)	13 (11.82%)
OSCE	66 (60.00%)	21 (19.09%)	22 (20.00%)
Shelf exam	84 (76.36%)	18 (13.36%)	8 (7.27%)

Medical student perceptions of assessment fairness. One missing survey in OSCE excluded

**Table 2:** Pairwise comparison of fairness

	Preceptor evaluations	Oral examination	Clinical documentation	OSCE	Shelf examination
**Preceptor evaluations**	–	< 0.0001*	< 0.0001*	0.0008*	< 0.0001*
**Oral examination**		–	0.3858	0.0152	0.2779
**Clinical documentation**			–	0.1629	0.0370
**OSCE**				–	0.0009*
**Shelf exam**					–

Pairwise comparison for fairness. Values in the table represent *p*-values. The null hypothesis is that the median of the population of differences between the paired data is zero (there is no difference in fairness between the two exams). Significance is 0.05/10 = 0.005. Significant values are marked with *

To investigate whether certain groups perceived assessment fairness differently, we performed subgroup analyses based on those who identified as URiM (Table 3), those who were planning on pursuing a surgical specialty (Table 4), and gender (Table 5). There were no statistically significant differences in perception of assessment fairness for any subgroup. Cliff’s delta, a measure of effect size, was consistent with *P*-values, with low effect size for the nonsignificant comparisons and moderate effect size for the significant comparisons.

**Table 3:** URiM subgroup analysis of fairness

Assessment	URiM (*n* = 19)	Non-URiM (*n* = 84)	*p*-value
Preceptor evaluations	7 (36.84%)	38 (45.24%)	0.5037
Oral examination	14 (73.68%)	64 (76.19%)	0.9181
Clinical documentation	10 (52.36%)	56 (66.67%)	0.4875
OSCE	12 (63.16%)	52 (61.90%)	0.4556
Shelf exam	15 (78.95%)	64 (76.19%)	0.7733

Subgroup analysis by those identifying as URiM. Percentage Strongly Agree and Agree reported. *P*-value is for the Wilcoxon Rank Sum analysis. Seven missing surveys excluded

Subgroup analysis for fairness of each assessment. Due to the number of tests, a multiplicity adjustment (Bonferroni Adjustment) was implemented, and the associated significance level of determination is now .05/5 = .01

**Table 4:** Surgical specialty subgroup analysis of fairness

Assessment	Pursuing surgical specialty (*n* = 27)	Pursuing non-surgical specialty (*n* = 81)	*p*-value
Preceptor evaluations	13 (48.18%)	33 (40.74%)	0.7707
Oral examination	19 (70.37%)	62 (76.54%)	0.4432
Clinical documentation	19 (70.37%)	50 (61.73%)	0.1898
OSCE	16 (59.26%)	50 (61.73%)	0.9418
Shelf exam	21 (77.78%)	63 (77.78%)	0.8668

Subgroup analysis by those who are planning on pursuing a surgical specialty. Percentage Strongly Agree and Agree reported. *P*-value is for the Wilcoxon Rank Sum analysis. Two missing surveys excluded

Subgroup analysis for fairness of each assessment. Due to the number of tests, a multiplicity adjustment (Bonferroni Adjustment) was implemented, and the associated significance level of determination is now .05/5 = .01

**Table 5:** Gender subgroup analysis of fairness

Assessment	Female (58)	Male (47)	*p*-value
Preceptor evaluations	24 (41.37%)	20 (42.55%)	0.8428
Oral examination	42 (72.41%)	36 (76.60%)	0.5252
Clinical documentation	41 (70.69%)	26 (55.32%)	0.0505
OSCE	33 (56.90%)	32 (68.09%)	0.4793
Shelf exam	44 (75.86%)	38 (80.85%)	0.7628

Subgroup analysis by gender. Percentage Strongly Agree and Agree reported. *P*-value is for the Wilcoxon Rank Sum analysis. Five missing surveys excluded

Subgroup analysis for fairness of each assessment. Due to the number of tests, a multiplicity adjustment (Bonferroni Adjustment) was implemented, and the associated significance level of determination is now .05/5 = .01

### Insight into clinical reasoning

Students were also asked whether they thought each assessment gave them insight into their clinical reasoning skills (Table 6). The preceptor evaluations remain the most poorly regarded assessment, with only 42.7% strongly agreeing or agreeing and 35.5% disagreeing or strongly disagreeing that they gave them insight into their clinical reasoning. The oral examination stands out as the most well-regarded assessment, with 80.0% of students strongly agreeing or agreeing that it gave them insight into their clinical reasoning. Additionally, the written feedback on the oral examination gave 77.3% of the group insight into their clinical reasoning. Our students also believed that the feedback on their clinical documentation gave them insight into their clinical reasoning (85.5% strongly agreeing or agreeing), though fewer students thought that writing the note gave them insight into clinical reasoning. (69.1% strongly agreeing or agreeing). The OSCE and feedback on the OSCE remained in the middle with 64.6 and 67.3% strongly agreeing or agreeing, respectively.

**Table 6:** Medical student perceptions of insight into clinical reasoning

Assessment	Strongly agree/Agree	Neutral	Disagree/Strongly disagree
Preceptor evaluations	47 (42.73%)	24 (26.32%)	39 (35.45%)
Oral examination	88 (80.00%)	11 (10.00%)	11 (10.00%)
Oral examination feedback	85 (77.27%)	18 (16.36%)	7 (6.36%)
Clinical documentation	76 (69.09%)	23 (20.91%)	10 (9.09%)
Clinical documentation feedback	94 (85.45%)	5 (4.54%)	11 (10.00%)
OSCE	71 (64.55%)	19 (17.27%)	20 (18.18%)
OSCE feedback	74 (67.27%)	20 (18.18%)	15 (13.64%)

Medical student perception of insight into clinical reasoning. One missing survey excluded from clinical documentation and OSCE feedback

To investigate each assessment’s ability to give students insight into their clinical reasoning, we performed pairwise comparisons of each assessment and their associated feedback as applicable. We found that fewer students significantly agreed or strongly agreed that the preceptor evaluations gave them insight into their clinical reasoning when compared to every assessment. On the other hand, the oral examination provided significantly more insight into clinical reasoning when compared to OSCE and OSCE feedback. The oral exam was rated higher than the OSCE, and oral exam feedback was higher than the OSCE and OSCE feedback. Clinical documentation feedback was significantly higher than clinical documentation for clinical reasoning skills. Clinical documentation feedback was significantly higher than the OSCE and OSCE feedback (Table 7). Again, Cliff’s delta was consistent with *P*-values, with low effect size for the nonsignificant comparisons and moderate effect size for the significant comparisons.

**Table 7:** Pairwise comparison of perceptions of insight into clinical reasoning

Assessment	Oral examination	Oral examination feedback	Clinical documentation	Clinical documentation feedback	OSCE	OSCE feedback
**Preceptor evaluations**	< 0.0001*	< 0.0001*	< 0.0001*	< 0.0001*	< 0.0001*	< 0.0001*
**Oral examination**	–	0.7835	0.0921	0.1526	0.0007*	0.0071
**Oral examination feedback**		–	0.0563	0.1950	0.0002*	0.0001*
**Clinical documentation**			–	< 0.0001*	0.0418	0.3693
**Clinical documentation feedback**				–	< 0.0001*	< 0.0001*
**OSCE**					–	0.1760

Pairwise comparison of perceptions of insight into clinical reasoning. The null hypothesis is the median of the population of differences between the paired data is zero. Significance is 0.05/21 = 0.0024. Significant values are marked with *

To investigate whether certain groups perceived the insight into clinical reasoning differently, we performed subgroup analyses based on those who identified as URiM (Table 8), those who were planning on pursuing a surgical specialty (Table 9), and gender (Table 10). There were no statistically significant differences in perception of insight into clinical reasoning for any subgroup.

**Table 8:** URiM subgroup analysis of clinical reasoning

Assessment	URiM (*n* = 19)	Non-URiM (*n* = 84)	*p*-value
Preceptor evaluations	10 (52.36%)	36 (42.86%)	0.6043
Oral examination	16 (84.21%)	67 (79.76%)	0.9667
Oral examination feedback	14 (73.68%)	66 (78.57%)	0.5928
Clinical documentation	11 (57.89%)	61 (72.62%)	0.5106
Clinical documentation feedback	15 (78.95%)	74 (88.10%)	0.5954
OSCE	15 (78.95%)	52 (64.29%)	0.4235
OSCE feedback	13 (68.42%)	59 (70.24%)	0.8481

Subgroup analysis by those who identified as URiM. Percentage Strongly Agree and Agree reported. *P*-value is for the Wilcoxon Rank Sum analysis. Seven missing surveys excluded

Subgroup analyses for student perceptions of insight into clinical reasoning. Due to the number of tests, a multiplicity adjustment (Bonferroni Adjustment) was implemented, the associated significance level of determination is now .05/5 = .01

**Table 9:** Surgical specialty subgroup analysis of clinical reasoning

Assessment	Pursuing surgical specialty (*n* = 27)	Not pursuing surgical specialty (*n* = 81)	*p*-value
Preceptor evaluations	12 (44.44%)	35 (43.21%)	0.8799
Oral examination	21 (77.78%)	67 (82.72%)	0.8368
Oral examination feedback	20 (74.07%)	64 (79.01%)	0.5520
Clinical documentation	20 (74.07%)	56 (69.14%)	0.5008
Clinical documentation feedback	23 (85.19%)	70 (86.42%)	0.7647
OSCE	16 (56.26%)	55 (67.90%)	0.6197
OSCE feedback	17 (62.96%)	57 (70.37%)	0.9939

Subgroup analysis for those pursuing a surgical specialty. Percentage Strongly Agree and Agree reported. *P*-value is for the Wilcoxon Rank Sum analysis. Two missing surveys excluded

Subgroup analyses for student perceptions of insight into clinical reasoning. Due to the number of tests, a multiplicity adjustment (Bonferroni Adjustment) was implemented, the associated significance level of determination is now .05/5 = .01

**Table 10:** Gender subgroup analysis of clinical reasoning

Assessment	Female (*n* = 58)	Male (*n* = 47)	*p*-value
Preceptor evaluations	24 (41.38%)	21 (44.68%)	0.8670
Oral examination	45 (77.59%)	40 (85.11%)	0.6471
Oral examination feedback	45 (77.59%)	37 (78.72%)	0.4044
Clinical documentation	40 (68.97%)	33 (70.21%)	0.5583
Clinical documentation feedback	50 (86.21%)	41 (87.23%)	0.1909
OSCE	37 (63.79%)	32 (68.09%)	0.4760
OSCE feedback	40 (68.97%)	32 (68.09%)	0.9583

Subgroup analysis for gender. Percentage Strongly Agree and Agree reported. *P*-value is for the Wilcoxon Rank Sum analysis. Five missing surveys excluded

Subgroup analyses for student perceptions of insight into clinical reasoning. Due to the number of tests, a multiplicity adjustment (Bonferroni Adjustment) was implemented, the associated significance level of determination is now .05/5 = .01

## Discussion

In this study, we found that the standardized shelf examination was the most highly regarded assessment with the oral examination a close second. Most students found the oral examination to be fairer than the preceptor evaluations and thought it gave them insight into their clinical reasoning compared to the preceptor evaluations and OSCE performance feedback (Tables 2 and 4). Our data support our hypothesis that medical students believe the oral examination is a fair and useful assessment tool on the general surgery clerkship. We did not find differences in the perception of fairness between URiM and non-URiM students nor males and females.

The reason for implementing our oral examination was to reduce bias and provide the clerkship director with objective data to guide their preceptor evaluation and final grades. Our data suggests the addition of an oral exam is well received by medical students. Though we anticipated our oral examination may be favored by URiM students like Caldwell et al., our cohort showed no statistically significant difference in URiM perceptions of any assessments, including the oral examination [7]. There was also no difference in fairness or insight into clinical reasoning based on gender or whether the student was pursuing a surgical specialty.

While oral examinations are not as widely used, preceptor evaluations of medical students’ performance often play a prominent role in the final clerkship grade, though they can be subjective and prone to bias [18, 19]. While there is a role for subjective evaluation of medical students by faculty to help assess traits that are unmeasured on standardized assessments, such as interpersonal skills, teamwork, work ethic, and leadership abilities, the literature has been contradictory as to whether these evaluations correlate with other measures of clinical knowledge. When used to evaluate clinical knowledge in a surgery clerkship, these evaluations often correlate poorly with other measures of clinical knowledge, such as standardized exams [9, 10]. Our study showed that students believe the preceptor evaluations deliver significantly less insight into clinical reasoning when compared to the oral examination, feedback on the oral examination performance, clinical documentation, feedback on clinical documentation, the OSCE, and feedback on OSCE performance. In addition to giving students limited insight into their clinical reasoning, preceptor evaluations seem to be poor differentiators of students; when students on a surgery clerkship were rated on a scale from 1 to 5 for overall clinical evaluation, with 3 being above-average performance and 5 being the top 15% of students, the average for all students was 4.4, with 86% rated greater than a 4 [10]. While preceptor evaluations can still provide valuable subjective insight into a student’s performance, they vary between preceptors and the literature suggests that they are not the most reliable method of determining clinical knowledge [10].

On the other hand, the OSCE is an examination that has been used for nearly 40 years that provides a valid, objective, comprehensive evaluation of students from both the evaluator and student’s perspective [20, 21]. The literature supports the use of OSCEs as a way to increase medical student perception into clinical reasoning [10, 22]. Luo et al. found that the use of an OSCE before a surgical clerkship increased medical student confidence immediately and after a month-long clerkship when compared to controls [22]. Furthermore, it has also been shown that medical students favor written feedback from the OSCE over other forms of feedback [23]. Our results are similar as our medical students thought both the OSCE and the written feedback gave them insight into their clinical reasoning abilities. While it offers value as a learning tool, the OSCE and their grading checklists may encourage test-taking behavior that is not necessarily representative of patient-centered care [24].

Another way to assess clinical reasoning is through clinical documentation. When clinical documentation is reviewed and scored according to differential diagnosis, justification, and workup, the scores from student notes correlate with clinical performance [25]. Additionally, Gagliardi et al. found that including medical students in actual documentation in the electronic health record led to increased opportunities for student teaching and feedback regarding student clinical reasoning. However, feedback rates from preceptors were significantly lower in procedural specialties like surgery [26]. Additionally, when evaluating the surgery clerkship, Dickinson et al. demonstrated that third-year medical students rate the importance of preceptor feedback as a 5 out of a 5-point scale but rated the actual frequency of this feedback as a 3 of 5 [27]. Our students also believed that the feedback on their patient notes gave them insight into their clinical reasoning. Overall, clinical documentation can provide insight into medical students’ clinical reasoning, and medical students desire feedback on their notes from their preceptors to maximize their education. However, there are barriers to providing note feedback in the general surgery clerkship, given the nature of the service.

There are several limitations to our study. First, this is a single institution study with a small sample size that limits our statistical power. Our sample size is particularly small for the URiM subgroup, making it difficult to assess. Regardless, the disparity undoubtedly faced by those who are URiM must be explored further to identify ways to provide fair assessment of all students on their general surgery clerkship. Our samples were also all from a single institution, which may not be representative of other institutions across the country. Our sample was a convenience sample, and therefore a power analysis was not performed. Other potential biases may include selection bias from our voluntary participation, response bias based on wording of the survey, and recency bias based on when participants took the survey relative to their assessments. Our goal is to make the grading system more equitable by using more objective assessments, but we acknowledge that there is still risk of some variation in student grades due to factors not controlled by students, such as rater variability, and inability to eliminate all bias. Finally, the self-perceived nature of this data is another major limitation, which limits our ability to draw conclusions based on this data alone.

Possible future directions include gathering data from future classes or other institutions to increase sample size and gathering perceptions of assessment fairness and insight into clinical reasoning from the clerkship director’s perspective. Comparing student perceptions with additional objective data, such as grades, would allow us to make stronger recommendations. Additional work is needed to investigate assessment methods more thoroughly to make grading systems more equitable for medical students. Despite these limitations, our study showed that the use of an oral examination during a medical school general surgery clerkship is fair according to medical student perceptions.

## Conclusion

While multiple assessment tools are used to evaluate performance on general surgery clerkships, medical students believe a structured, case-based oral examination is a fair assessment. In addition, significantly more medical students agree that it offers them insight into their clinical reasoning abilities compared to other assessments. However, more work is needed to identify and eliminate unjust grading metrics to create an equitable grading system for medical student clinical clerkships.

## Data Availability

The dataset generated and analyzed during this study are available from the corresponding author on reasonable request.

## Abbreviations

OSCE: Observed standardized clinical encounter
NBME: National board of medical examiners
AAMC: Association of American Medical College
EPA: Entrustable professional activities
URiM: Underrepresented in medicine
